# *Borrelia burgdorferi* initiates early transcriptional re-programming in macrophages that supports long-term suppression of inflammation

**DOI:** 10.1371/journal.ppat.1011886

**Published:** 2023-12-29

**Authors:** Tanja Petnicki-Ocwieja, Julie E. McCarthy, Urmila Powale, P. Kent Langston, Jennifer D. Helble, Linden T. Hu

**Affiliations:** 1 Department of Molecular Biology and Microbiology, Tufts University School of Medicine, Boston, Massachusetts, United States of America; 2 Graduate Program in Immunology, Tufts Graduate School of Biomedical Sciences, Boston, Massachusetts, United States of America; 3 Department of Immunology, Harvard Medical School and Evergrande Center for Immunologic Diseases, Harvard Medical School and Brigham and Women’s Hospital; Boston, Massachusetts, United States of America; University of Montana, UNITED STATES

## Abstract

*Borrelia burgdorferi (Bb)*, the causative agent of Lyme disease, establishes a long-term infection and leads to disease manifestations that are the result of host immune responses to the pathogen. Inflammatory manifestations resolve spontaneously despite continued bacterial presence, suggesting inflammatory cells become less responsive over time. This is mimicked by *in vitro* repeated stimulations, resulting in tolerance, a phenotypic subset of innate immune memory. We performed comparative transcriptional analysis of macrophages in acute and memory states and identified sets of Tolerized, Hyper-Induced, Secondary-Induced and Hyper-Suppressed genes resulting from memory induction, revealing previously unexplored networks of genes affected by cellular re-programming. Tolerized gene families included inflammatory mediators and interferon related genes as would be predicted by the attenuation of inflammation over time. To better understand how cells mediate inflammatory hypo-responsiveness, we focused on genes that could mediate maintenance of suppression, such as Hyper-Induced genes which are up-regulated in memory states. These genes were notably enriched in stress pathways regulated by anti-inflammatory modulators. We examined one of the most highly expressed negative regulators of immune pathways during primary stimulation, Aconitate decarboxylase 1 (Acod1), and tested its effects during *in vivo* infection with *Bb*. As predicted by our *in vitro* model, we show its inflammation-suppressive downstream effects are sustained during *in vivo* long-term infection with *Bb*, with a specific role in Lyme carditis.

## Author summary

Lyme disease, caused by the spirochete *Borrelia burgdorferi (Bb)*, is a long-term infection whose inflammatory manifestations are primarily due to an exuberant immune response to the pathogen. Although *Bb* can persist in its hosts for prolonged periods, most patients will resolve their symptoms even without antibiotic treatment. This suggests that the host has evolved methods of dampening the inflammatory response. Tolerance is a phenotypic subset of innate immune memory, a response resulting from epigenetic changes induced by a primary stimulus or during an acute infection. This transcriptional memory can be retained, affecting how a person responds during long-term infection or to subsequent infections. To better understand the gene expression changes between acute and memory states, we used an *in vitro* model of repeated stimulations and performed RNA sequencing. We identified a complex transcriptional profile network involved in the suppression of inflammatory responses and revealed novel gene expression subsets and pathways that may be important in maintenance of memory as well as gene sets responsible in the induction of memory during primary stimulation. We focused on one such gene, Acod1, a negative regulator of inflammation, and show that its anti-inflammatory effects are maintained in an animal model of *Bb* infection.

## Introduction

*Borrelia burgdorferi (Bb)* is an important human pathogen that causes almost 500,000 cases per year in the U.S. [1] *Bb* is maintained in wildlife reservoirs including small rodents and birds [2]. Once infected, the natural hosts are thought to remain infected for life. Interestingly, infection causes little to no signs of disease in its wildlife hosts despite high levels of bacteria throughout multiple tissues including skin, heart, bladder and spleens [2]. In non-native hosts like humans, infection can cause symptoms including the characteristic erythema migrans rash, carditis, meningitis and arthritis [2]. However, these symptoms can resolve even in the absence of treatment, although in some patients bacteria can still be detected months to years after the initial infection [3]. The reasons for a lack of an inflammatory response despite the presence of bacteria are not well understood.

Previous studies have suggested that the innate immune system plays a critical role in early detection and response to the bacterium. Initial *Bb* recognition occurs primarily by engagement of toll-like receptor (TLR) 2 with additional contributions by TLR5,7,8,9, nucleotide binding oligomerization domain containing (NOD) 2, and Nod-like receptor protein (NLRP)3 [4–9]. During *in vitro Bb* stimulations, as well as in direct injection models where macrophage are exposed to the organism for the first time, deficiencies in these receptors result in expected decreases in activation of inflammatory responses. However, in long term murine infection models, loss of many of these receptors or related molecules results in increased inflammation [8–10]. While the loss of these receptors sometimes resulted in a decreased ability to control infection and increase bacterial loads, loss of others that did not affect bacterial clearance (e.g. NOD2 and TIR-domain-containing adaptor inducing interferon β (TRIF)) also resulted in increased inflammation, suggesting that the increase in inflammation may not be explained by increased bacteria alone [8,11]. One possibility is that disruption of innate pathogen recognition and response during primary microbial encounter may result in a failure to trigger compensatory suppressive mechanisms that are important for sustaining long-term tolerance to persistent infection.

One mechanism by which this immunoregulation could occur is through innate immune tolerance, a phenotypic subset of innate immune memory that has emerged as an important concept in describing a transcriptional memory resulting from a primary infection [12,13]. During repeated stimulation with the same type of ligand, a previous induction renders the inflammatory genes less responsive to subsequent stimulus, becoming tolerized, a phenomenon well described in *in vitro* sepsis models using lipopolysaccharide (LPS) or endotoxin [14]. Notably, tolerance is often discussed with respect to gut homeostasis where it is beneficial for the immune system to limit its response to the continued presence of commensal microbiota [15–17]. While the exact mechanisms governing innate immune tolerance are not well understood, past studies have noted that primary stimulation with microbial ligands results in cellular reprogramming characterized by epigenetic and metabolic changes, which drive the phenomenon [12,13,18,19]. Transcriptional memory in peripheral myeloid cells is not long lasting due to rapid cell turnover; however, there is now increasing evidence that hematopoietic stem progenitor cells can acquire memory upon pathogen infection, which renders innate immune memory more long lasting than previously recognized [12,13,20,21].

Induction of innate immune memory during Lyme disease could result in the suppression of inflammation during long-term infection, allowing for resolution of inflammation and symptomatic recovery. We hypothesized that infection with *Bb* results in the reprogramming of the innate immune system to prevent inflammation associated with long term infection. We explored this hypothesis by utilizing an *in vitro* model of repeated *Bb* stimulations in bone marrow derived macrophages (BMDMs).

Using RNA sequencing (RNAseq), we identify a complex innate immune memory transcriptional profile and describe novel gene expression sets and pathways of interest that could drive long-term inflammatory suppression. We specifically investigate *Acod1* as relevant in memory induction and demonstrate an *in vivo* role during long-term infection with *Bb*. Our studies show that Acod1 has tissue specific consequences on inflammation during murine Lyme carditis. This work characterizes the re-alignment of the immune response over time in response to *Bb* and highlights the *in vivo* impact of disrupting transcriptional memory in the context of Lyme disease.

## Results

### RNA sequencing reveals complex differential gene expression in *B*. *burgdorferi* induced memory

**Fig 1:**
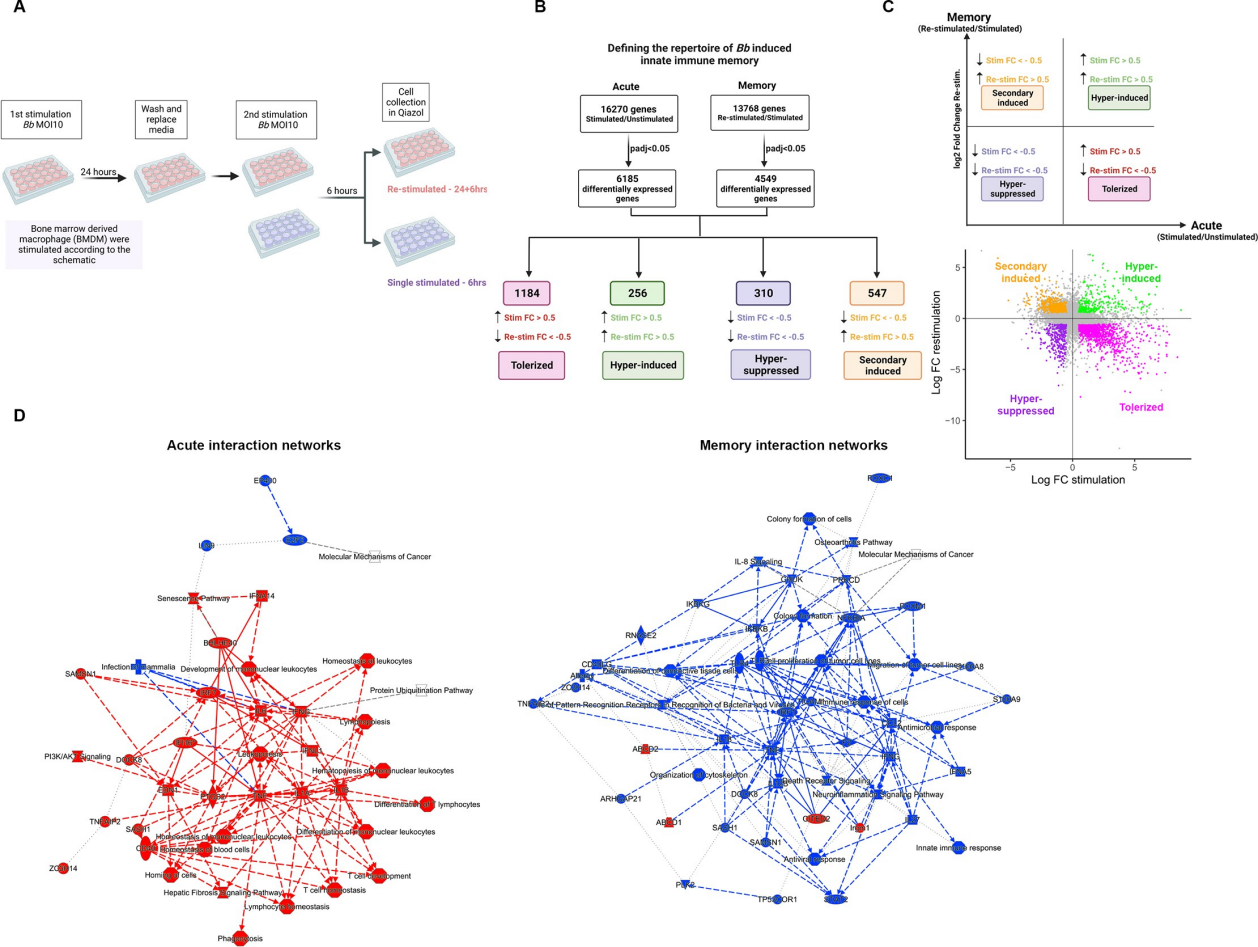
RNA sequencing reveals complex differential gene expression in *Bb* induced memory. **A)** Experimental workflow of induction of memory in macrophage. Memory macrophages were stimulated with *Bb* (MOI 10) for 24 hours, washed, then re-stimulated for 6 hours. Memory BMDMs were compared to BMDMs stimulated for 6 hours (primary or acute). Drawn with Biorender. **B)** Flowchart of the differential expression analysis for stimulation and re-stimulation experiments. An adjusted *P* value (Padj) cutoff of <0.05 was used to define differentially expressed genes (DEGs). Data from both experiments were compared and genes were categorized as tolerized, hyper-induced, secondary induced, based on log2 Fold Change (with a cutoff of >0.5 or <-0.5) in each experiment. Drawn with Biorender. **C)** DEGs for stimulation and re-stimulation experiments. Individual dots represent DEGs. The x-axis represents log2FC for the stimulation experiment compared to unstimulated (primary or acute); the y-axis represents log2FC for the re-stimulation experiment compared to the stimulated (memory). Drawn with Biorender and R software. **D)** Ingenuity Pathway Analysis (IPA) network of the primary (acute) and memory data sets. Activated pathways are colored in red and inhibited pathways are colored in blue. Red and blue lines between nodes represent activation or inhibition, respectively. Dotted lines are inferred relationships and dashed lines are indirect interactions.

In order to model the changes in immune responses during long-term infection, we performed bulk RNAseq from BMDMs during single and repeated stimulation. To do so, we stimulated bone marrow derived BMDMs for 24 hours with *Bb*, washed the cells and then did a secondary stimulation with *Bb* for 6 hours. In parallel, naïve bone marrow derived BMDMs were also left unstimulated as well as stimulated for 6 hours as a comparison. Samples single stimulated for 6 hours were labeled as “stimulated” and represent a primary response that can be thought of as acute due to the significant pro-inflammatory response that is rapidly induced. Cells that were stimulated for 24 hours followed by a 6 hour stimulation were labeled as “re-stimulated” and represent the memory response (Fig 1A). This differs from a previous study of repeated *Bb* stimulations but is consistent with studies of endotoxin tolerance suggesting that macrophage have entered a tolerant state by 24 hours and highlights the importance of comparisons with early time points [19,22,23].

Differential expression analysis of the RNA sequencing of the stimulated compared to unstimulated BMDMs tested 16,270 genes. Using an adjusted p-value (padj) cutoff of <0.05, there were 6,185 differentially expressed genes (DEGs). Differential expression analysis of the re-stimulated BMDMs compared to the stimulated BMDMs tested 13,768 genes. Using the same padj cutoff of <0.05, there were 4,549 DEGs (Fig 1B). We used an additional cutoff of log2 fold change (log2FC) of less than -0.5 and greater than 0.5, eliminating genes that did not undergo any major changes in gene expression upon primary or secondary stimulation. Of the statistically significant genes, Tolerized genes (n = 1,184) were defined as having a log2FC >0.5 in the stimulation experiment and a log2FC <-0.5 in the re-stimulation experiment. Hyper-Induced genes (n = 256) were defined as having a log2FC >0.5 in the stimulation and re-stimulation experiments. Secondary Induced genes (n = 547) were defined as having a log2FC <0.5 in the stimulation experiment and a log2FC >0.5 in the re-stimulation experiment. Hyper-Suppressed genes (n = 310) were defined as having a log2FC <0.5 in both the stimulation and re-stimulation experiments (Fig 1B and 1C)

Genes were plotted as individual dots based on the log2FC in a scatter plot (Fig 1C). The four quadrants correspond to the different gene expression categories identified in our analysis: Tolerized (describing genes that were up-regulated in primary and down-regulated in re-stimulation), Hyper-Induced (up-regulated in both stimulations), SecondaryInduced (down-regulated upon stimulation and up-regulated in re-stimulation), and hyper-suppressed (down-regulated in both stimulations). An Ingenuity Pathway Analysis (IPA) of primary/acute and memory interaction networks showed that the most represented pathways were immune related and were activated during exposure of naïve cells. These pathways were inhibited in memory samples, suggesting that there is a down-regulation of inflammatory pathways upon re-stimulation, consistent with a tolerant innate immune state (Fig 1D).

### Repeated *B*. *burgdorferi* stimulations tolerize pro-inflammatory signaling

**Fig 2:**
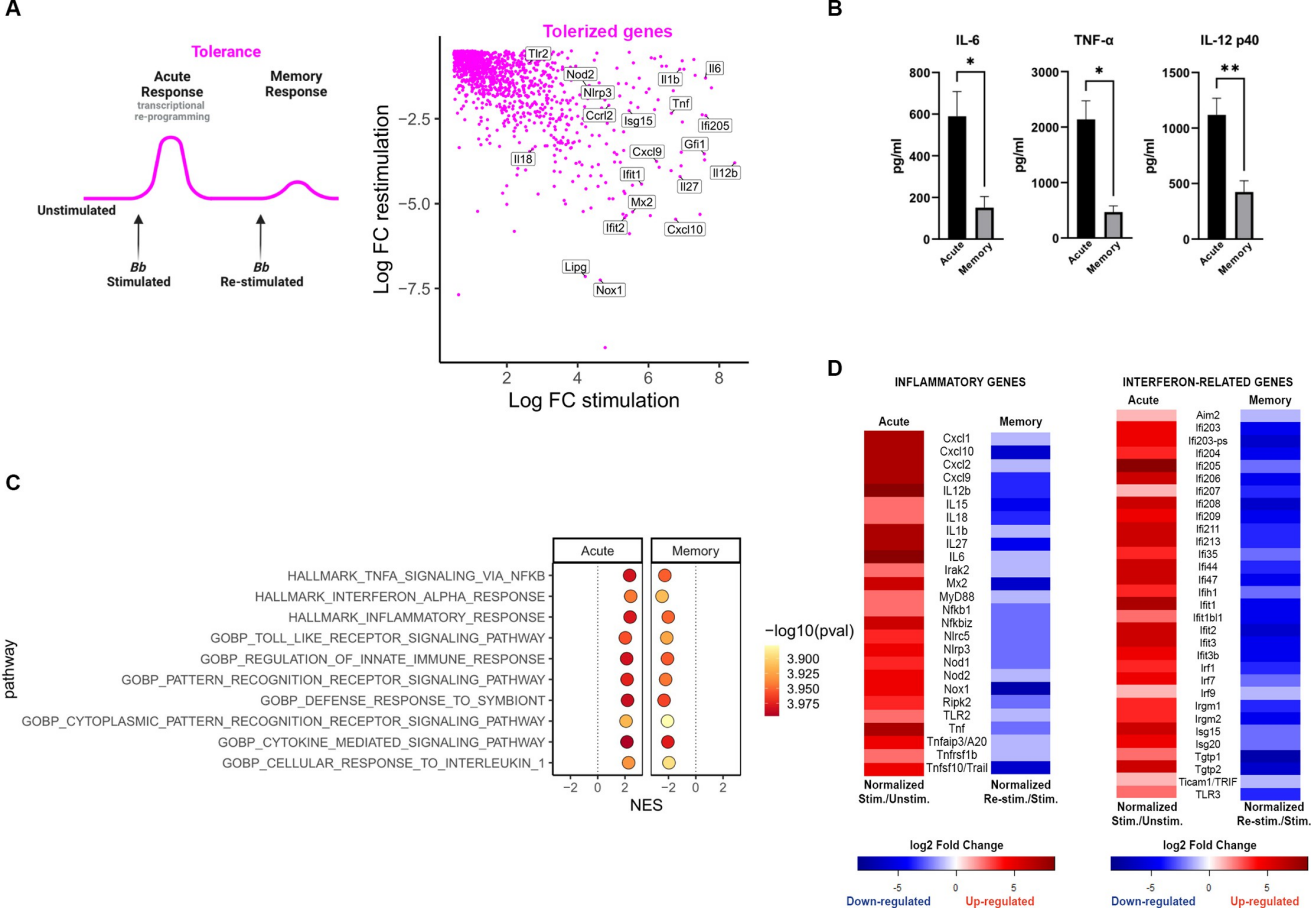
Repeated *Bb* stimulation tolerizes pro-inflammatory signaling. **A)** Model of primary (acute) and memory changes in gene expression in inflammatory tolerance (drawn with Biorender), accompanied by a scatter plot of Tolerized genes that were up-regulated in the primary (acute) and down-regulated in memory compared to primary stimulation. Genes of interest are noted. **B)** Bone marrow derived macrophage (BMDMs) were stimulated with *Bb* (MOI 10) for both acute and memory timepoints. Supernatants were collected and cytokine levels of IL-6, TNF-α and IL-12 p40 were quantified by ELISA. Data is from three independent experiments, analyzed by ratio paired T-test, *p<0.05. **C)** Ten of the top tolerized pathways from Gene Set Enrichment Analysis (GSEA). Tolerized gene sets are defined as having a Normalized Enrichment Score (NES) >0 for the stimulation and a NES <0 for the re-stimulation experiment. All shown gene sets have p-values <0.05 for both the stimulation and re-stimulation. **D)** Heatmap of inflammatory and interferon related DEGs in the tolerance quadrant in the primary (acute) and memory conditions. Red indicates up-regulation, blue indicates down-regulation. Intensity is based on log2FC.

Numerous studies have described innate immune tolerance, characterized by an acute inflammatory response upon initial stimulation and a muted memory response during repeated stimulation [12,14,22,24–26]. Tolerized genes can thus be described as those that are up-regulated upon stimulation compared to unstimulated but down-regulated upon repeated stimulation compared to the primary response. This indicates a hypo-responsiveness of inflammatory mediators in the tolerized cells, characterized by a down-regulation of innate immune receptors, innate immune signaling molecules and inflammatory pathways through epigenetic and metabolic rewiring [14,19,25,27]. Confirming that *Bb* mediated tolerance mirrors previously described gene expression changes in other systems, our results showed that pro-inflammatory mediators such as *tnf-α*, *il6*, *il1β*, *il12*, *cxcl10* were among the most differentially expressed in the tolerance category (Fig 2A). Protein expression for a subset of these genes was confirmed by ELISA (Fig 2B). Gene Set Enrichment Analysis (GSEA) of the Tolerizedgenes identified 581 different pathways, among which the top hits represented well characterized TNF, interferon (IFN) and pattern recognition receptor (PRR) pathways (Fig 2C). We further analyzed gene expression of known inflammatory mediators and found an expected subset of genes known to signal downstream of *Bb* (Fig 2D). Of interest were the large numbers of type I interferon related genes in this Tolerized category. In previous studies, *in vitro* measurements of type I IFNs by ELISA or qPCR have not shown a very robust response to single *Bb* stimulation [7,28]. However, during *in vivo Bb* infection, type I IFNs have been shown to be important in the development of Lyme arthritis in a mouse model [29]. Our data confirms that despite previous low levels of detection, *Bb* indeed induces a robust and varied type I IFN response critical in the induction of inflammation (Fig 2D). Additionally, several genes that were highly differentially expressed were of interest due to their unique role in inflammatory responses (Table 1). Data from our RNAseq experiment shows that the pro-inflammatory cytokine and interferon responses are induced during the initial contact with *Bb* and are down-regulated following repeated stimulations, thus providing a mechanism for limiting long-term inflammation.

**Table 1:** Tolerized genes of interest.

				Stim/Unstim		Restim/Stim	
Gene	Gene Name	Immune related function		Log2fold change	padj	Log2fold change	padj
Gbp10	Guanylate-binding protein 10	GTPase involved with vesicular trafficking in host defense [79]		6.70	3.31E-05	-1.67	0.05
Gbp5	Guanylate binding protein 5	Dynamin-like GTPase that is an activator of NLRP3 inflammasome [53]		6.93	5.09E-60	-3.49	3.96E-235
Ifi205	Interferon activated gene 205	AIM2-like receptor involved in DNA sensing [80]		7.62	3.61E-173	-2.41	1.48E-72
Ptgs2 (Cox2)	Prostaglandin-endoperoxide synthase 2	Cyclooxygenase enzyme which suppresses dendritic cell immunity [81]		7.32	6.27E-90	-1.22	8.46E-30

### Hyper-Suppressed and secondary induced genes offer novel insights into cellular re-programming

**Fig 3:**
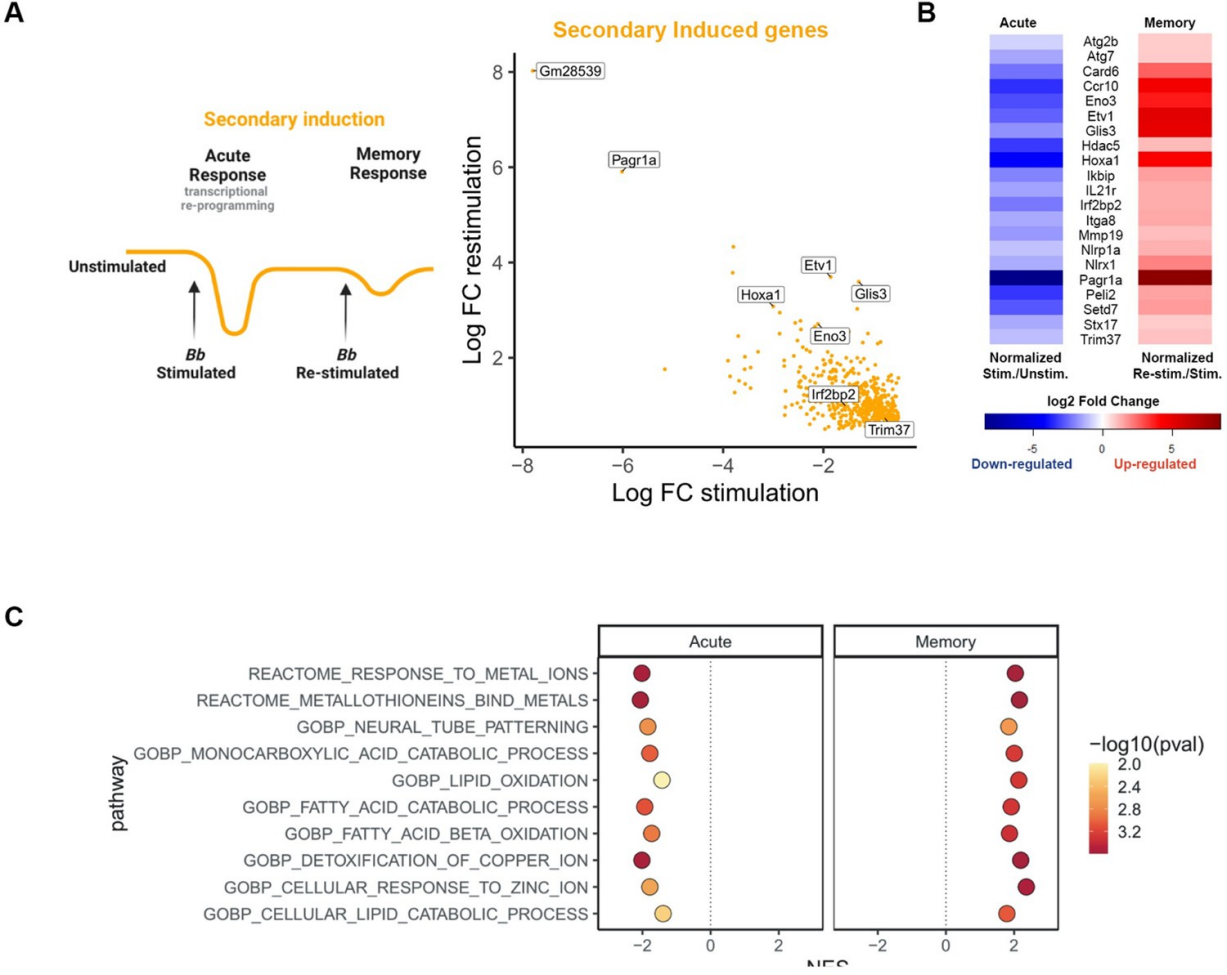
Secondary induced genes are present in lipid oxidation and catabolism pathways. **A)** Model of primary (acute) and memory changes in gene expression in Secondary Induction (drawn with Biorender), accompanied by a scatter plot of Secondary Induced genes that were down-regulated in the primary (acute) and up-regulated in memory compared to primary stimulation. Genes of interest are noted. **B)** Heatmap of Secondary Induced DEGs of interest in primary (acute) and memory conditions. Red indicates up-regulation, blue indicates down-regulation. Intensity is based on log2FC. **C)** Ten of the top secondary induced gene sets from GSEA. Secondary induced gene sets were defined as having a NES <0 for the stimulation and NES >0 for the re-stimulation experiment. All shown gene sets have p-values <0.05 for stimulation and re-stimulation.

As part of our analysis, we identified genes that are down-regulated upon primary stimulus. Of particular interest are genes that were down-regulated in macrophages stimulated for the first time, but up-regulated in the re-stimulated samples, which we term the Secondary Induced genes (Fig 3). These genes are specifically up-regulated during the memory response and thus might be important in regulating long term immunosuppression. From our analysis, the top ten GSEA pathways included Response to Metal Ions and Lipid Oxidation and Catabolic processes. Genes that fit this expression pattern often encode proteins involved in catabolism and have been shown to be immunosuppressive through poorly defined mechanisms, such as Glis3 and Hoxa1 (Fig 3B, 3C and Table 2) [30,31].

**Table 2:** Secondary induced genes of interest.

				Stim/Unstim		Restim/Stim	
Gene	Gene Name	Immune related function		(Log2fold change)	padj	(Log2fold change)	padj
Ccr10	CC Motif Chemokine Receptor 10	A chemokine receptor that regulates T cells responsiveness in epithelial immunity [82]		-2.45	0.1	3.25	0.01
Glis3	GLIS Family Zinc Finger 3	Transcription factor Susceptibility gene for type I and type II diabetes [83,84]		-1.29	2.81E-05	3.60	4.44E-63
Hoxa1	Homeobox A1	Transcription factor Plays a role in controlling microbial diversity in the intestine [85] Responsible for regulation of immunosuppressive molecules [31]		-3.00	0.01	3.08	0.02
Pagr1a	PAXIP1 associated glutamate rich protein 1A	Histone methyltransferase complex involved in transcriptional regulation of B-cell switch regions [86]		-6.02	2.21E-10	5.91	2.14E-09

**Fig 4:**
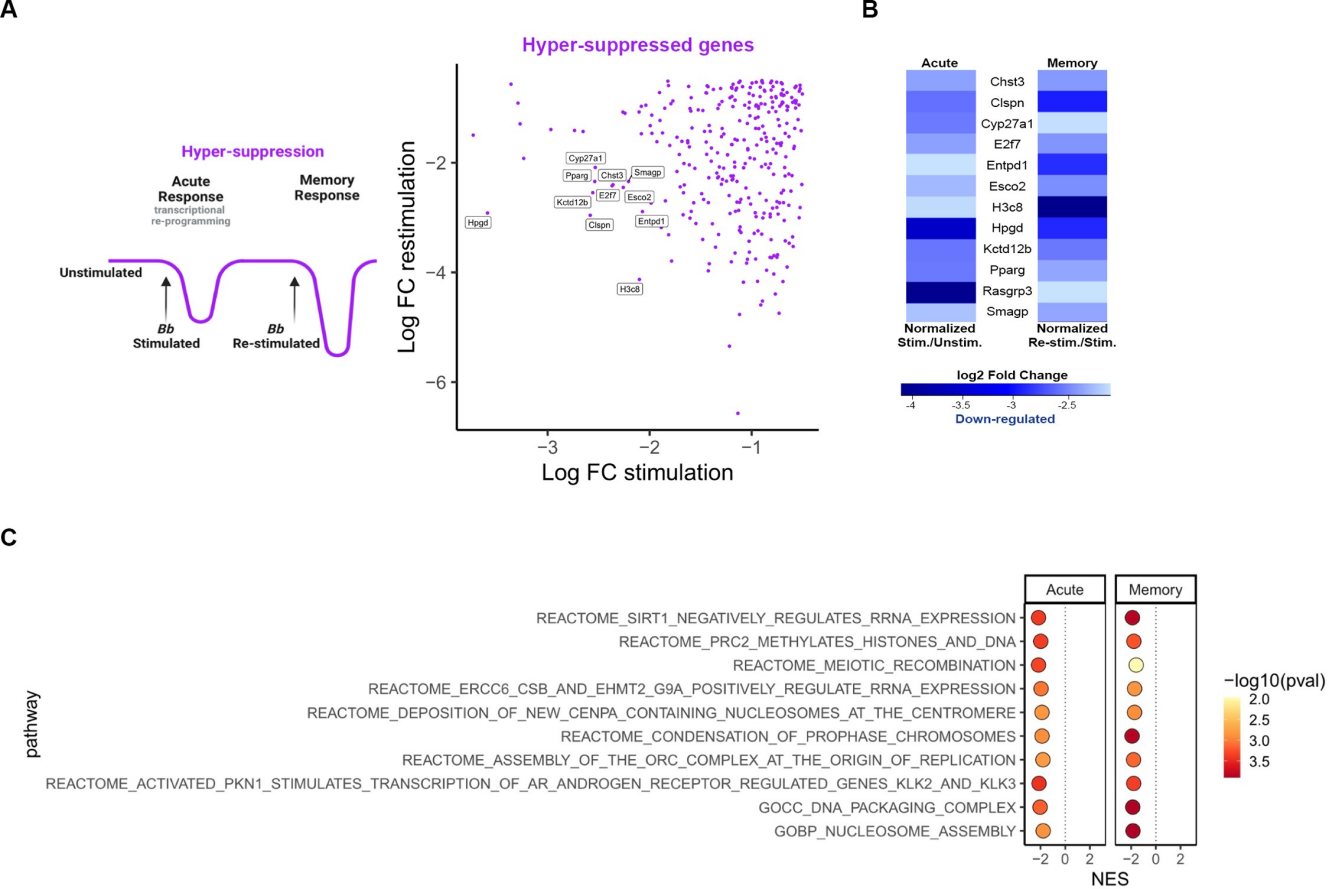
Genes involved in general cell maintenance are down-regulated during infection. **A)** Model of primary (acute) and memory changes in gene expression in Hyper-suppression (drawn with Biorender), accompanied by a scatter plot of Hyper-suppressed genes that were down-regulated in the primary (acute) and down-regulated in memory compared to primary stimulation. Genes of interest are noted. **B)** Heatmap of Hyper-suppressed DEGs of interest in primary (acute) and memory conditions. Blue indicates down-regulation. Intensity is based on log2FC. **C)** Ten of the top hyper-suppressed gene sets from GSEA. Hyper-suppressed gene sets were defined as having a NES <0 for the stimulation and NES <0 for the re-stimulation experiment. All shown gene sets have p-values <0.05 for stimulation and re-stimulation.

Hyper-Suppressed genes are down-regulated in both the first stimulation and further down-regulated in the memory response. Our analysis showed that a number of the top ten GSEA pathways had roles in gene regulation and nucleic acid maintenance. These genes may be of interest as they may be important cellular function pathways that are down-regulated to divert energy to host defense and are thus critical in maintaining long term homeostasis (Fig 4A–4C). Future studies are needed to determine the interplay between general cellular pathways and defense pathways in order to coordinate an efficient response to pathogen infection.

### Cellular stress pathways that may mediate long-term immunosuppression are enriched in Hyper-Induction

**Fig 5:**
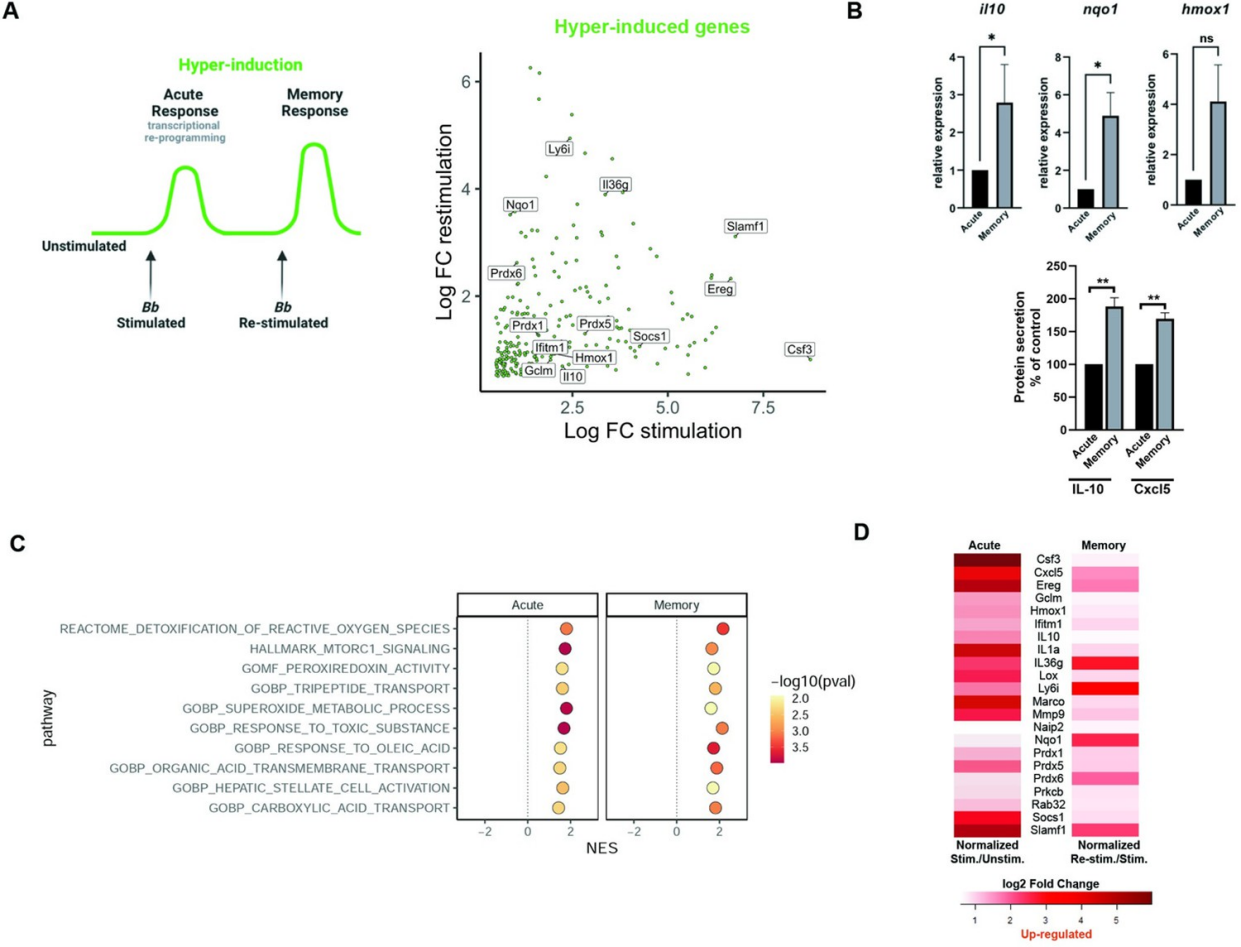
Hyper-induced genes are represented in a number of stress pathways. **A)** Model of primary (acute) and memory changes in gene expression in Hyper-induction (drawn with Biorender), accompanied by a scatter plot of Hyper-induced genes that were up-regulated in the primary (acute) and further up-regulated in memory compared to primary stimulation. Genes of interest are noted. **B)** BMDMs were stimulated with *Bb* (MOI 10) for both primary (acute) and memory timepoints. RNA was collected and gene expression levels of *il10*, *nqo1* and *hmox1* were quantified by RT-qPCR. Expression was normalized to the primary stimulation. Data is from four independent experiments analyzed with Mann-Whitney U test. IL-10 and Cxcl5 were measured by ELISA from supernatants of BMDM stimulated at 6hrs (acute) and 24+6hrs (memory). Stimulated cells (acute) were compared to unstimulated controls. Data is graphed as mean percent change +/- SEM in memory states compared to acute from two independent experiments and analyzed by unpaired T test, *p<0.05, **p<0.01.**C)** Ten of the top hyper-induced gene sets from GSEA. Hyper-induced gene sets are defined as having a NES >0 for the stimulation and re-stimulation experiments. All shown gene sets have p-values <0.05 for both the stimulation and re-stimulation. **D)** Heatmap of Hyper-induced DEGs of interest in primary (acute) and memory conditions. Red indicates up-regulation. Intensity is based on log2FC.

Trained immunity is used to describe hyper-induction in gene expression during the memory response. Here we identified genes showing hyper-induction upon re-stimulation and may mediate long-term immunosuppression (Fig 5A). Some anti-inflammatory mediators have been shown to fit this hyper-induced profile, suggesting they are important in limiting inflammation long-term. One such mediator is IL-10, a known negative regulator of inflammation and necessary for driving tolerance to LPS (Fig 5B and 5C) [14]. In addition, the Cxcl5 neutrophil recruiting chemokine, shown to play a role in suppression of sepsis, was identified as having a hyper-induced phenotype (Fig 5B) [32]. We identified a number of different known or possible mediators of inflammation that may play a role in regulating the memory response (Fig 5B and 5C). Our GSEA analysis of Hyper-Induced genes identified 30 pathways, of which the top 15 pathways were primarily related to detoxification of reactive oxygen species as well as other toxic compounds (Fig 5D). During long-term infection there is a likely buildup of toxic compounds, and removal is critical for proper cellular function. An example of Hyper-Induced detoxification genes includes *prdx6* (Fig 5C and Table 2), or perioxiredoxin 6, represented in the top GSEA identified pathway, together with *prdx5* and *prdx1*.

Of particular interest were genes such as *nqo1* and *hmox1* which appeared repeatedly in the top fifteen Hyper-Induced GSEA pathways related to oxidative stress and detoxification (Table 3). Both genes are anti-oxidant genes regulated by Nrf2, which suppresses inflammatory mediators such as IL-6 and IL1β [33,34]. By qPCR, we confirmed that *nqo1* is a hyper-induced gene and determined that *hmox1* is a re-inducible gene (Fig 5B).

**Table 3:** Hyper-induced genes of interest.

				Stim/Unstim		Restim/Stim	
Gene	Gene Name	Immune related function		Log2fold change	padj	Log2fold change	padj
Ifitm1	Interferon induced transmembrane protein 1	Transmembrane protein that restricts entry by a variety of viruses including SARS-Cov2 [87]		1.77	0.03	1.15	2.40E-06
Nqo1	NAD(P)H Quinone Dehydrogenase 1	Cytoplasmic electron reductase induced via Nrf2 to respond to oxidative stress and suppress inflammation [33,34] Inhibits Ikbz and suppresses TLR mediated cytokine induction [60]		0.87	0.01	3.52	1.16E-107
Prdx6	Peroxiredoxin 6	Peroxidase involved in detoxification of peroxides during oxidative stress [58]		1.05	2.86E-14	2.62	2.35E-180
IL-36g	Interleukin 36 gamma	Can promote wound healing in response to TLR3 [88] Loss of IL36g receptor results in increased IBD inflammation [89]		3.36	1.12E-08	3.89	9.58E-79
Hmox1	Heme oxygenase 1	Cyclooxygenase enzyme induced via Nrf2 to respond to oxidative stress and suppress inflammation [33,34]		2.04	3.08E-62	0.92	6.68E-24

### Acod1 is highly up-regulated in primary infection

After characterizing *Bb* induced primary and memory responses in macrophages, we investigated how the transition from the primary to memory responses occurs over time. Negative regulators of inflammation, such as the anti-inflammatory cytokine IL-10, have been shown to be key in the establishment and maintenance of tolerance [14]. In order to up-regulate genes that are important in long term immunosuppression, many negative regulators are induced during later phases of acute stimulation.

**Fig 6:**
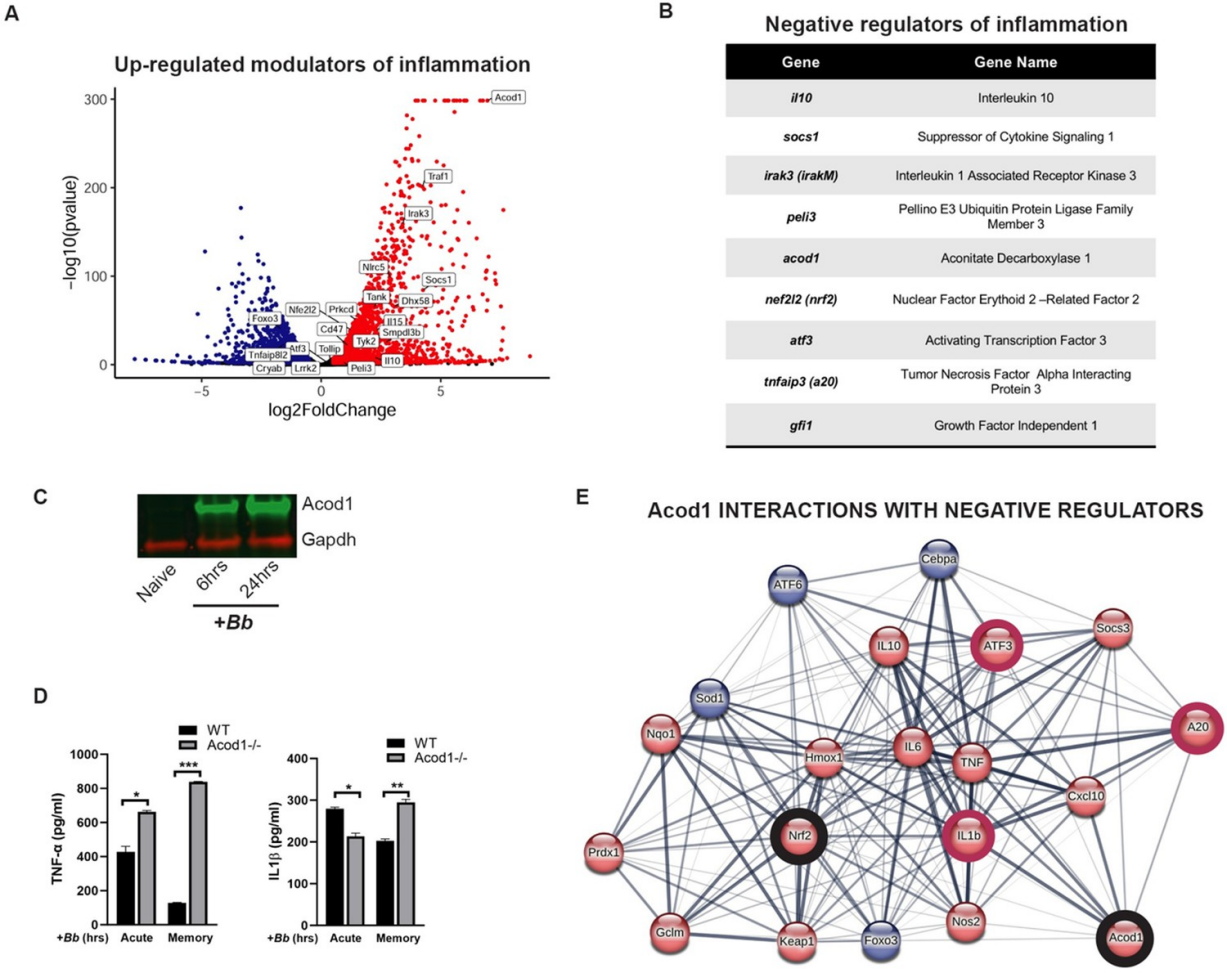
Acod1 is a highly up-regulated modulator of inflammation. **A)** Volcano plot of DEGs in primary stimulation. Described negative regulators of inflammation are noted. **B)** Table of genes with known roles in negative regulation of innate immune responses found in our stimulated data set. **C)** BMDMs were stimulated with *Bb* (MOI 10) for 0, 6 and 24 hours. Cells were collected and protein expression levels of Acod1 were assessed by immunoblot with a Gapdh loading control. Data are representative of two experiments. **D)** BMDM were stimulated for 24 hours or 48 hours (TNFα and IL1β respectively) with *Bb* MOI 100 and 2 hours 5mM ATP. Cells were washed and re-stimulated with *Bb* MOI 10 for 10hrs and 2 hrs 5mM ATP. TNFα and IL1β were measured by ELISA. Graph represents the mean +/-SEM of two independent experiments analyzed by T-test, **p<0.01, ***p<0.001. **E)** Genes from the Ingenuity Pathway Analysis of stimulated genes that identified Acod1 and Nrf2 as upstream regulators were loaded into the STRING interaction network. Red indicates up-regulation, blue indicates down-regulation. Acod1 and Nrf2 are highlighted as well as negative regulators A20 and ATF3 and IL1β as a target gene.

Therefore, we assessed the expression of known negative regulators of innate immune signaling and inflammation upon primary stimulation (Fig 6A). *Bb* induced the up-regulation of numerous negative regulators including *il10*, s*ocs-1*, *pellino 3* and others [14,35,36]. Among those induced by *Bb* we also identified *nfe2l2* (*nrf2*), which was consistent with the identification of *nqo1* and *hmox1* in the Hyper-Induced gene set (Fig 6A and 6B).

The transcript for *acod1* was among the most highly up-regulated negative regulators identified (log2FC 6.95; p <0.001) (Fig 6A and 6B). Acod1 (Aconitate decarboxylase 1) is an enzyme that converts cis-aconitate to the metabolite itaconate. Itaconate is known to have a variety of immunosuppressive functions including activation of anti-inflammation transcription factors Nrf2 and Atf3 [27,37,38]. Our RNAseq data showed a strong induction in *acod1* expression in a primary stimulation which we confirmed at the protein level using anti-Acod1 antibodies (Figs 6C and S2). We show that during *Bb* stimulation, Acod1 production remains up-regulated over time, showing a high level of protein expression as early as 6 and 24 hours. Because the effects of Acod1 on cytokine expression have varied in different studies [38,39], we chose to first establish a time course of cytokine secretion in wild-type versus Acod1 deficient BMDM stimulated with *Bb*. In order to measure IL1β, we stimulated BMDM for 10, 24 and 48 hours with *Bb* MOI 10 and 2 hrs of 5mM ATP. Simultaneously, we measured TNFα and IL6 and found that not only did Acod1 have a differential effect on cytokines but that its effects were also dependent on cytokine kinetics (S3 Fig). Acod1 effects on TNFα were observed at 10hrs post-stimulation, while IL1β showed opposite effects at 10hrs versus 48hrs. IL6 did not show any significant differences between wild-type and Acod1 deficient cells. As a result, we chose to test stimulations (acute) and re-stimulations (memory) at 10hrs and 24+10hrs for TNFα, and 10hrs and 48+10hrs for IL1β (Fig 6D). Both TNFα and IL1β resulted in increased secretion in Acod1 deficient cells compared to wild-type in memory states, suggesting Acod1 plays a role in suppressing inflammation to long term *Bb* stimulation. Using data from our IPA analysis we created a network using the STRING interaction database to visualize the links between Acod1 and other negative regulators such as Nrf2, Atf3 and A20 (Fig 6D), suggesting Acod1 may be a master negative regulator of inflammation.

### Acod1 contributes to the suppression of murine Lyme carditis

**Fig 7:**
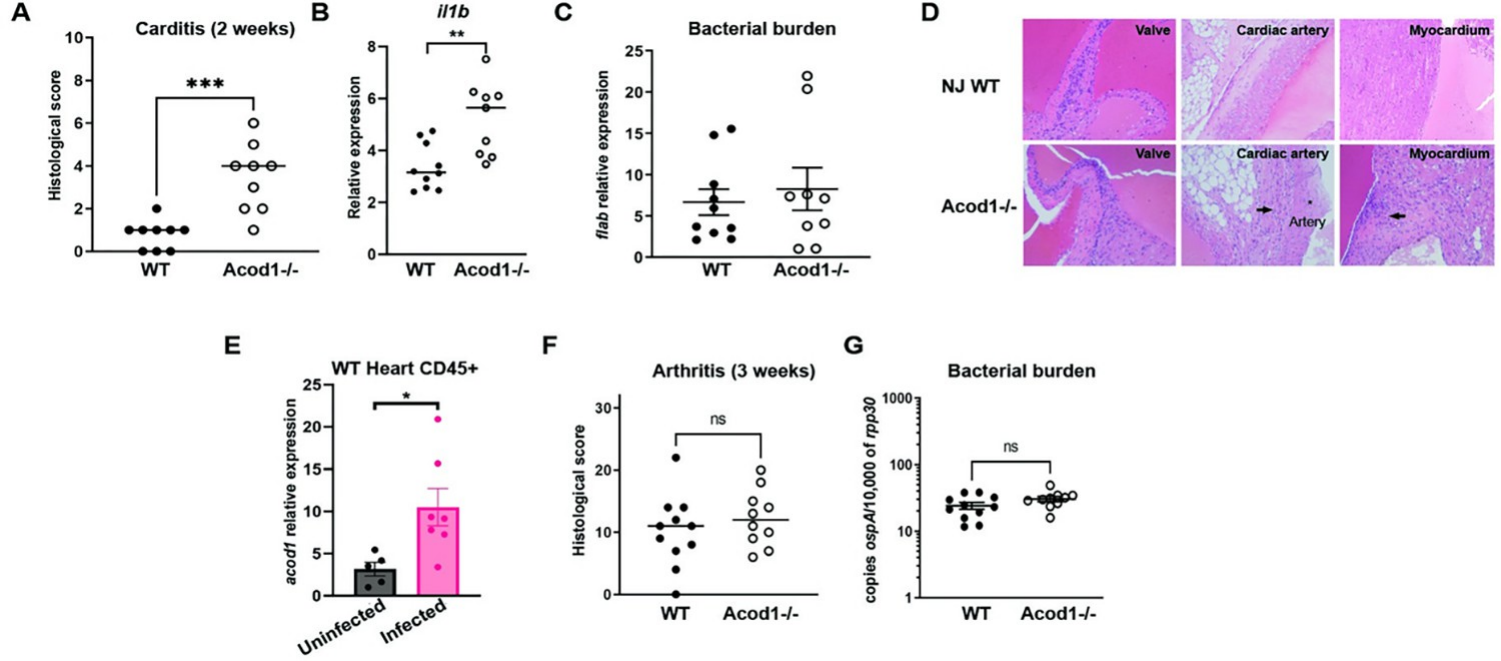
Acod1 is important in the regulation of Lyme carditis *in vivo*. **A)** Two weeks post infection hearts from NJ WT and Acod1^-/-^ mice were sectioned and stained with H&E and scored blindly by a pathology for inflammation using a scale of 0–10. Nine mice from two independent experiments were analyzed with Mann-Whitney Test. **B)** Two weeks post infection hearts from NJ WT and Acod1^-/-^ mice were homogenized in Qiazol and RNA was isolated. Gene expression of *il1β* were normalized to uninfected controls and quantified by qPCR. Three mice were used for the BSK controls and nine-ten mice were used for infection from three independent experiments analyzed by Mann-Whitney U. **C)** Bacterial loads were quantified from heart cDNA, two weeks post infection, by qPCR for bacterial *flab*, normalized to mouse *hprt* and analyzed by Mann Whitney U. **D)** Representative heart H&E stained sections from two week infected NJ WT and Acod1-/- mice are shown for inflammation in the valve, cardiac artery and myocardium. **E)** Two weeks post *Bb* infection or control hearts from NJ WT were homogenized and CD45+ cells isolated using a microbead purification per manufacturer’s instructions. Enriched leukocytes were resuspended in Qiazol and RNA was isolated. Gene expression of *acod1* was quantified by qRT-PCR. 5 mice used for BSK controls and 7 for Bb infected and analyzed by Mann-Whitney U, *p<0.05. **F)** Hind limbs from three week infected mice in two independent experiments were sectioned and stained with H&E and scored blindly by a pathologist for inflammation using a scale of 0–32. Ten-Eleven mice from three independent experiments were analyzed with Mann-Whitney Test. **G)** Bacterial loads were quantified from bladder DNA by ddPCR with bacterial probe OspA and mouse reference probe rpp30 and analyzed by unpaired -test.

Based on our *in vitro* results, we next investigated whether *Acod1* played an appreciable role in Lyme immunopathology in the joints and heart where infected animals show inflammation. To assess the role of Acod1 in Lyme carditis, which peaks at two weeks post infection in wild type mice [40,41], we subcutaneously infected Acod1^-/-^ and C57BL/6NJ WT mice and terminated at two weeks. The bacterial load was measured from either cDNA of heart tissue or from bladder DNA. It has been previously shown that spirochete burden in the bladder are consistent with loads in other tissues including in our study where we did not observe differences in bacterial burdens in the heart (Fig 7E and 7C) [8–10,42–44]. Bacterial burdens of the Acod1^-/-^ mice are unchanged compared to WT two or three weeks post infection, suggesting that the increase in inflammation is not due to increased bacterial burden. Hearts were scored based on leukocyte infiltrate (Fig 7A). Histological analysis of the hearts indicated that Acod1^-/-^ exhibited increased inflammation in comparison to WT mice. Although WT infected mice did exhibit some inflammation in heart tissue, Acod1-/- mice had statistically significant increases in lesion severity compared to WT, exhibiting periarteritis, myocarditis and valvulitis (Fig 7A and 7D). Given these results, we processed whole hearts for RNA to examine which Acod1 target genes are up-regulated in a *Bb* infection and of those which may be contributing to increased inflammation in the Acod1^-/-^ mice. We found that *il1β* was significantly up-regulated in the Acod1^-/-^ compared to their WT counterparts (Fig 7B). To assess if immune cells were responsible for driving the phenotype in *Bb* infected heart tissue, we isolated CD45+ cells and identified a significant up-regulation of *acod1* expression in these cells from *Bb* infected mice compared to controls (Fig 7D).

We also assessed the presentation of Lyme arthritis in both Acod1^-/-^ and WT mice by collecting joints for histological analysis of arthritis at three weeks post infection and extracted DNA from the bladder for bacterial loads. Acod1^-/-^ mice did not display significantly increased inflammation in the joints or in bacterial burden in comparison to their WT counterparts (Fig 7E and 7F). Together, our data suggests that prolonged *Bb* infection results in induction of Acod1 which is necessary to suppress Lyme carditis, but not arthritis. These data suggest that Acod1 is a regulator of anti-inflammatory responses important in the memory response to *Bb* induced carditis.

## Discussion

*B*. *burgdorferi* establishes a life-long asymptomatic infection in its mouse reservoir host [45,46]. The lack of inflammation despite the presence of bacteria in tissues suggests that the immune system may enter a state of suppression where it is purposefully blinded to the organism. This may benefit both the bacteria and the host in a situation where most of the damage is caused by activation of the immune system. In non-reservoir host inbred mice, the infection causes an inflammatory response during the acute phase of infection, followed by an anti-inflammatory phase and symptom resolution [9,42,47,48]. This is also observed in the natural history of human infection where most people will resolve their acute inflammatory symptoms even without antibiotic treatment. The innate immune system has evolved a series of checks on persistent inflammation in response to repeated exposures to a stimulus. These have been most prominently explored for gut commensal bacteria where prior exposures will alter subsequent cellular responses. A notable difference with *Bb* is that, rather than being contained in the gut, the organism disseminates throughout the body resulting in multiple potential exposures to different parts of the immune system [2]. We hypothesized that resolution of inflammation despite persistent infection is the result of innate immune memory altering responses to the organism and that over time, the immune system treats *Bb* as an “invasive commensal organism”. Our goal was to investigate mechanisms of memory using RNAseq in an *in vitro* tolerance model of *Bb* infection.

We chose a model of repeated stimulation that has been used effectively in endotoxin studies to predict immune responses in sepsis, favoring a shorter length of stimulation for the acute time point, 6hrs versus the 16–20 hrs used for *Bb* in Barriales et al [23]. We observed differences between our study and the Barriales et al. data in the magnitude of differential expression (such as TNFα, IL1β, Ifi205, Socs1, Hoxa1 and others) or the directionality of gene expression between acute and memory states (Cxcl10, Ifit1, Irf7, Cxcl5 and others). Recent studies with LPS indicate that the acute phase of stimulation is shorter than many studies have previously indicated, and that induction of metabolic and epigenetic changes that lead to innate immune memory occur early. By 12–24 hours post LPS stimulation, cellular re-programming is already underway, and cells shift to a tolerant state [19]. Our study adds new information comparing memory states to early acute time points in order to identify early regulators of memory.

In our analysis, we illustrated that pro-inflammatory mediators are Tolerized genes. This is consistent with the attenuation of inflammation after acute infection leading to symptom resolution in animals and humans. Within the Tolerized gene set, we identified a broad initial type I IFN inducible transcriptional induction which then resolved on repeat stimulation, again consistent with previous reports that type I IFNs are required for the induction of murine Lyme arthritis *in vivo* [49–51]. In addition to commonly studied pro-inflammatory mediators we identified other genes of interest in the Tolerized gene set for their less well-known roles in inflammatory responses, such as Gbp5, a guanylate binding protein (GBP). GBPs are dynamin related GTPases which are induced by type I IFNs and pro-inflammatory cytokines [52]. Although described as a trafficking molecule, Gbp5 also seems to have a pro-inflammatory role in activating NLRP3 inflammasome assembly, thus allowing NLRP3 to respond to stimuli [53]. As a pro-inflammatory mediator in response to acute infection, its suppression in memory states is consistent with our other observations.

An additional aspect of our study was the analysis of genes that are suppressed, rather than induced, upon acute stimulation. These categories are often overlooked in other studies. We describe them in two separate groups: Hyper-Suppressed (further down-regulated in memory) and Secondary Induced (up-regulated in memory). Hyper-Suppressed genes fell into pathways describing general cellular mechanisms with no direct links to immunity. The reason for down-regulation of these genes is not clear, but the clustering of multiple genes suggests that it is not random. One possible hypothesis is that while under the stress of pathogen invasion there is an energetic reshuffling within the cell towards pathways important in host defense rather than cellular division and chromosome maintenance. In contrast, many of the SecondaryInduced genes had potential links to immune regulation. For example, the Hoxa1 transcription factor is responsible for increased levels of immunosuppressive molecules such as arginase 1 and the transcription factor STAT3 [31]. This is intriguing, as Hoxa1 and a number of other Secondary Induced genes do respond to microbial stimulus but not at the time or in the manner we would expect. They are not only induced exclusively in memory states, but also, rather than inducing inflammatory signals, seem to be immunosuppressive instead. It is tempting to speculate that the cellular re-programming that occurs during acute infection results in a transcriptional memory that primes immunosuppressive genes which are then ready to curtail inflammation in response to a second infection. Genes in the Secondary Induced category largely have not been explored in the context of long-term microbial infections or innate immune memory and further studies are warranted to understand the role of these novel genes and pathways in repeated stimulations.

Our study offers additional insights into previously described non-tolerized gene subsets, such as those we categorize as Hyper-Induced (up-regulated in primary and further up-regulated in memory responses). We reasoned that this set of differentially expressed genes would likely be mediators of anti-inflammatory long-term maintenance due to their hyper-responsiveness to the initial stimulus. Interestingly, we did not find a strong enrichment of known anti-inflammatory cytokines and mediators, besides IL-10. IL-10 has been shown to mediate the subsequent down-regulation of the TLR4 receptor and phagocytic receptors as well as up-regulation of other negative regulators of TLR signaling, such as the kinase Irak3/IrakM [14,26,54–56]. In the context of *Bb* infection, IL-10 deficient mice exhibit significantly higher arthritis severity compared to wild type [57]. We also identified an enrichment in pathways that participate in toxic substance and reactive oxygen species detoxification. One potential explanation for this may be that they are critical to controlling cellular alarms to continued stimulation by limiting the accumulation of Danger Associated Molecular Patterns (DAMPs) that activate innate immune pathways but also have a dual role in suppressing inflammatory signals directly. However, it is also possible that once specific responses to infection are induced during the acute phase, pathways that are important during long-term infection are less specific and are general stress pathways that mediate a return to homeostasis and reduce inflammation. Importantly, an early induction of these genes may be critical in establishing the appropriate immunosuppressive mechanisms long-term, as suggested by the induction of Hyper-Induced genes in the acute phase of infection. Examples of Hyper-Induced molecules include Cxcl5, a neutrophil chemoattractant that has been shown to limit the development of sepsis. However, an exact mechanism for how Cxcl5 participates in innate immune memory has not been described[32]. Additional examples include Prdx6, a detoxification enzyme which catalyzes the reduction of hydrogen peroxide and organic hydroperoxides to water [58], as well as Prdx1 which negatively regulates NF-kB activity by interfering with the TRAF6 and mitochondrial ECSIT complex. The ECSIT complex isinvolved in the production of mitochondrial reactive oxygen species [59]. The activity of Prdx1 as a negative regulator of inflammation would be consistent with the hypothesis that these genes mediate immunosuppression long-term by remaining up-regulated. In addition to the peroxiredoxin family of *prdx* genes, we found that anti-oxidant genes such as *nqo1* and *hmox1* are also Hyper-Induced. Similarly to Prdx, Nqo1 promotes degradation of IkBz important in the TLR mediated induction of pro-inflammatory cytokines [60]. As such, Nqo1 also negatively regulates inflammatory responses, suggesting that a number of the Hyper-Induced genes are immunosuppressive.

Expanding our search of negative regulators induced upon *Bb* stimulation, we identified *acod1* as very highly induced during primary infection. Acod1 controls the production of itaconate, a metabolite that was first discovered as a negative regulator of oxidative metabolism by inhibiting succinate dehydrogenase [61,62]. In recent years, *acod1* has been shown to be up-regulated in a variety of infection models [63–67]. Itaconate has also been implicated in innate immune memory, both training and tolerance [27,68]. Through itaconate production, Acod1 is thought to induce the transcription factor Nrf2, which directly promotes Nqo1 and Hmox1 anti-oxidant signaling and suppressing pro-inflammatory cytokines like IL-6, IL-12 and IL-1β [27,33,34,37]. These studies provide a link between Acod1/Nrf2 and the subset of anti-oxidant and anti-inflammatory genes we identify in our Hyper-Induced subset. However, Acod1, through itaconate, suppresses inflammation through the activation of other transcription factors such as Atf3 and affects a variety of pathways, including glucose metabolism, which have immunosuppressive effects, specifically affecting only IL1β secretion in some studies and IL6 in others [38,39]. That Acod1 may be an early trigger of suppressive reprogramming of inflammation is consistent with our finding that *acod1* was highly induced by *Bb* in the acute phase, as early as 6 hours post infection. In contrast to LPS stimulation, a time course of *Bb* stimulation revealed an increased level of secretion in Acod1 deficient cells for TNFα and IL1β. In restimulation experiments, *Bb* stimulated Acod1 deficient BMDM showed increased secretion for both IL1β and TNFα, compared to wild-type cells in memory states. Therefore, Acod1 may contribute to the induction of innate immune memory, as an early induced master negative regulator of inflammation.

We show that the immunosuppressive effects of Acod1 were maintained, over time, in a mouse infection model. We identified a role for Acod1 in the resolution of Lyme carditis, showing that Acod1 deficiency leads to amplification of *il1β* expression in the heart and ultimately increased Lyme carditis assessed by histopathology. This inflammatory phenotype was independent of bacterial burden, as we showed no change in bacterial loads in Acod1 deficient mice compared to wild type. This suggests that the increased pathology in the Acod1^-/-^ mice is not a consequence of itaconate’s known anti-microbial mechanism of inhibiting bacterial isocitrate lyase, a conclusion further bolstered by predictive genetic analysis that *Bb* lacks citric acid cycle enzymes [69–71]. It is note-worthy that the immune consequences of an Acod1 deficiency are tissue specific, primarily affecting carditis but not arthritis. Lyme carditis is driven primarily by macrophage infiltration, and therefore itaconate dependent immunosuppression may be specifically mediated by macrophages compared with arthritis which is neutrophil driven [40]. Notably, Dominguez et al., demonstrated that a single nucleotide polymorphism in Acod1 could affect human monocyte IL-6 and TNF tolerance to *Bb* [27].

Our studies highlight the complex interaction between *Bb* and host immunity that evolves over time with repeated exposure. Early inflammation gives way to down-regulation of inflammatory processes through a complex, interconnected network of regulators. We have shown that the effects of repeated exposure *in vitro* on negative regulators of inflammation appear to parallel those seen *in vivo* during infection. The role of many of the genes that are identified as differentially regulated during initial and subsequent exposure remain to be determined and understood. Studies have shown that even individual single nucleotide polymorphisms may significantly affect responses to *Bb* and down-regulation of these responses [72]. By better understanding how responses to *Bb* are down-regulated over time with exposure, we may also better understand what has gone awry in cases where inflammatory responses are not appropriately attenuated, which is postulated to be a potential mechanism for patients who continue to have symptoms after treatment for Lyme disease [3].

## Materials and methods

### Ethics statement

All mice were housed in the Tufts University Animal Facility until use. All experiments were approved by the Tufts Institutional Animal Care and Use Committee (IACUC, Protocol #B2021-84). Euthanasia was performed using CO_2_ asphyxiation followed by cervical dislocation in accordance with guidelines from the American Veterinary Medical Association (AVMA) and was approved by the Tufts IACUC.

### Generation of Bone Marrow Derived Macrophages (BMDMs)

BMDMs were generated as previously described [73]. Briefly, bone marrow cells were flushed from mouse femurs and tibiae with sterile DMEM and cultured on 100 mm x 15 mm plastic petri dishes for 5–7 days in DMEM media supplemented with 30% L929 cell conditioned medium, 20% FBS and 1% penicillin-streptomycin.

### Infection of bone marrow derived macrophage cultures

2x10^6^ BMDM were plated per well in 6 well plates and stimulated. *Bb* were washed three times with DMEM with 10% FBS, counted and resuspended in the same media. Media from BMDMs was removed and replaced with the same media containing *Bb* at an MOI of 10. Cells were harvested by collecting the cell culture supernatant and adding TRIzol (Invitrogen) to the remaining cells for RNA extraction analysis. For Immunoblotting, cells were lysed in RIPA buffer with protease inhibitor cocktail and heat denatured at 95°C in sample buffer.

### RNAseq processing & differential gene expression

Libraries were prepared and sequenced on the Illumina HiSeq platform by Genewiz Inc at a sequencing depth at approximately 25 million reads per-sample. Paired-end RNASeq reads were aligned to the mm10 mouse reference genome using STAR version 2.5.3a [74] and gene expression was summarized using RSEM version 1.3.0 [75]. All differential gene expression analyses were performed with R (version 4.0.2) using DESeq2 version 1.30.1 [76]. Genes with less than 10 reads total across all samples were removed from the analysis. When comparing experimental conditions, the input model was gene ~ stim where stim was a factor with the two conditions being compared (unstimulated vs. stimulated or stimulated vs. re-stimulated). A Wald test was then used to calculate P values and a false discovery rate (FDR) was computed using the Bejamini-Hochberg method. Genes with an FDR < 0.1 were considered significant. All significant DEGs can be found in S1 Table. Graphical scatter plots and heat maps were created using R software.

### Gene set enrichment analysis, Ingenuity Pathway Analysis and STRING network

Gene set enrichment analysis (GSEA) was performed using the fgsea package (version 1.16.0) with 10000 permutations for independence. The input gene rankings for all analyses were the Wald-statistic values when comparing conditions, where the gene with the highest Wald statistic was ranked first and the lowest Wald statistic was ranked last. Gene sets tested included those in the HALLMARK, KEGG, GOBP, GOMF and REACTOME libraries. Tolerized gene sets were defined has having a Normalized Enrichment Score (NES) >0 for the stimulation and NES<0 for the re-stimulation. Hyper-induced gene sets were defined as having a NES >0 for both the stimulation and re-stimulation experiments. Secondary induced gene sets were defined as having a NES <0 for the stimulation and NES >0 for the re-stimulation. Hyper-suppressed gene sets were defined as having a NES >0 for the stimulation and re-stimulation experiments. All fgsea results are available in S2 Table. Additional pathway analysis was performed using Ingenuity Pathway Analysis (IPA) with a padj cutoff of <0.1 and a log2FoldChange cutoff of |0.5|. Selected hits from the IPA analysis of “Up-stream regulators” in the Acod1 and Nrf2 pathways were used as an input into the STRING protein-protein interaction network (string-db.org) using the *Mus musculus* database. Lines linking proteins were set to a confidence of 0.150 in an interactive display mode showing query protein names. Node coloring mode enabled to color activated proteins in red and suppressed proteins in blue.

### Mouse and bacterial strains

Acod1 deficient mice (Jackson 029340) and C57BL/6NJ (Jackson), C57BL/6J (Jackson) mice were housed in specific pathogen free rooms according to the Tufts Institutional Animal Care and Use Committee.

An infectious clone of *B*. *burgdorferi* B31 or N40 was used for all stimulations or infections. For *in vitro* experiments, *B*. *burgdorferi* was cultured in Barbour-Stoenner-Kelley (BSK) [71] complete media at 37°C to early stationary phase, and the cell density was determined using a Petroff-Hauser counting chamber. Bacteria were used at a multiplicity of infection (MOI) 10:1 in all experiments. For mouse infections, 1x10^5^ B31 was injected subcutaneously.

### Antibodies and reagents

Samples for Immunoblot were prepared according to Bambouskova et al. [38] Immunoblot studies were performed using the indicated antibodies: anti-Gapdh 1:500 (Santa Cruz, 25778), anti-Actin 1:500 (Cell Signaling), anti-Acod1 (Cell Signaling, 17805) 1:500 and visualized on LICOR.

### RNA isolation and quantitative analysis

To perform qPCR analysis of transcripts, BMDM cells or whole hearts were collected in Qiazol or Trizol RNA was extracted using Qiagen miRNAeasy kit following the manufacturer’s instructions. Contaminating DNA was removed using the Qiagen DNAse free kit. RNA was resuspended in water. cDNA was synthesized from up to 1 μg of RNA measured by spectrophotometer using the ImPromII kit (Promega) following the manufacturer’s instructions. DNA contamination within the RNA template sample was avoided by using primers spanning introns. Cycling parameters for SYBR Green-based reactions were 95°C for 15 minutes, 40 cycles of 95°C for 30 seconds, 60°C for 30 seconds, and 72°C for 30 seconds, followed by 95°C for 1 minute, 55°C for 1 minute. The amount of target, normalized to an endogenous reference (HPRT) and relative to a calibrator, is given by 2− ΔΔCt, where Ct is the cycle number of the detection threshold. Primers sequences are as follows: *hprt* (F: 5’-GTTAAGCAGTACAGCCCCAAA-3’, R: 5’-AGGGCATATCCAACAACAAACTT-3’), [73], *hmox1* (F: 5’-CACTCTGGAGATGACACCTGAG-3’, R: 5’-GTGTTCCTCTGTCAGCATCACC-3’) (origene.com), *nqo1* (F: 5’-AATGGGCCAGTACAATCAGG-3’, R: 5’-CCAGCCCTAAGGATCTCTCC-3’)[77], *il10* (F: 5’-AGAGCTGCGGACTGCCTTCA-3’, R: 5’-AATGCTCCTTGATTTCTGGG-3’) [11], *flab* (F: 5’-TTGCTGATCAAGCTC AATATAACCA-3’, R: 5’-TTGAGACCCTGAAAGTGATGC-3’).

### ELISA

Supernatants were collected from BMDM cultures at 6 hours and 24+6 post stimulation, unless indicated. Cytokines were measured by ELISA using the IL-6 kit, IL12p40 (R&D Systems), TNF-α (R&D Systems) or the Cxcl5 Duo Set kit (R&D Systems) following the manufacturer’s instructions.

### Droplet digital PCR

Mouse bladders were isolated at specific time points post-infection and DNA was extracted using the DNeasy Blood and Tissue Kit (Qiagen). Droplet digital PCR (ddPCR) was used to quantify the ratio of Borrelia ospA to mouse rpp30 using ddPCR Supermix for Probes (no dUTP) (Bio-Rad) with the following primers and probes (5’ ➔ 3’): ospA fwd: ATGTTAGCAGCCTTGACGAG; ospA rev: TCGTACTTGCCGTCTTTGTT; ospA probe: 6-FAM-AGCGTTTCAGTAGATTTGCCTGGTG-Iowa Black FQ; rpp30 fwd: CCAGCTCCGTTTGTGATAGT; rpp30 rev: CAAGGCAGAGATGCCCATAA; rpp30 probe: HEX-CTGTGCACACATGCATTTGAGAGGT-Iowa Black FQ. Oil-for-Probes droplet emersions were generated using the QX200 droplet generator (Bio-Rad). PCRs were run for 10 minutes at 95°C, 40 cycles of 94°C for 30 seconds and 60°C for 1 minute, followed by 10 minutes at 98°C and subsequent cooling to 4°C. Fluorescent reads were calculated using QX200 droplet reader, analyzed using QX Manager Software (Bio-Rad), and reported as a ratio of copies of *Borrelia* ospA per 10,000 copies of mouse rpp30 [78].

### CD45+ cell enrichment from heart tissue

Hearts were perfused with 1xPBS and incubated in 0.9mg/ml of collagenase for 20 minutes. A cell suspension was created by passing heart tissue through a 40 micron cell filter. CD45+ cells were isolated by incubating with CD45 selection microbeads (Miltenyi biotech) in MACS buffer (1xPBS pH 7.2, 0.5% FBS, 2mM EDTA) and passing through an LS column (Miltenyi biotech) per manufacturer’s instructions. After selection, cells were resuspended in Qiazol for RNA isolation.

### Heart and joint histology

Heart and joint were isolated from WT and Acod1^-/-^ mice at specified time points post-infection. Tissues stored in Bouins before being paraffin embedded, sectioned and stained with hematoxylin and eosin (H&E) at the Rodent Histopathology Core at Harvard Medical School. Slides were blindly scored by the Tufts Veterinary Pathologist using the Lyme disease scoring system outlined from Drs. Denise Imai and Kelly Ramsey at University of California at Davis. Joints were scored based on synovitis, tenosynovitis/tendonitis, periarticular fibrosis, synovial proliferation, articular cartilage degeneration or erosion, periosteal remodeling, and effusion/edema severity. Hearts were scored on myocarditis, valvulitis, arteritis, myocardial fibrosis severity.

### Statistical analysis

Data were analyzed using Graphpad Prism software. Differences were considered statistically significant if the P value was less than 0.05. For all figures, * P < 0.05, ** P < 0.01, and *** P < 0.001.

### Graphical models and drawings

All graphical representations were created in Biorender.

## Supporting information

S1 Table Differential gene expression analysis for stimulation vs unstimulated conditions (acute) and re-stimulation vs stimulation conditions (memory). Results include gene name (gene), normalized transcript counts averaged for all samples (baseMean), the log2 fold-change estimate between the conditions being compared (log2FoldChange), standard error of the log2FoldChange estimate (lfcSE), Wald-statistic (stat), *P* value (pvalue), adjusted *P* value (padj), and the comparison performed (contrast).
(XLSX)

S2 Table Gene set enrichment analysis results with each row representing a pathway (pathway), corresponding *P* value (pval), adjusted *P* value (padj), enrichment score (ES), normalized enrichment score (NES), number of times a random gene set had a more extreme enrichment score value (nMoreExtreme), number of genes from the pathway found in our data set (size), leading edge genes that drive the enrichment score (leadingEdge), and comparison performed (contrast). (XLSX)

S1 Fig Principal Component Analysis of RNAseq. Principal component analysis of RNAseq data. Samples are colored by treatment of BMDMs: none (n = 2), stimulation (n = 2), re-stimulation (n = 2).
(TIFF)

S2 Fig Acod1 is induced upon *Bb* stimulation. BMDMs were stimulated with *Bb* (MOI 10) for 0, 6 and 24 hours. Cells were collected and protein expression levels of Acod1 were assessed by immunoblot with β-actin as a loading control.
(TIFF)

S3 Fig Cytokine time-course in *Bb* stimulated Acod1-/- cells. Bone marrow derived macrophage from NJ WT and Acod1 deficient mice were stimulated with *Bb* MOI10 for 10, 24 and 48 hours. Supernatants were collected at each time point and cytokines measured by ELISA. Graphed are two independent experiments and statistical significance assessed by unpaired T test, *p<0.05.
(TIFF)
