# Supplementary figures and images for: Borrelia burgdorferi initiates early transcriptional re-programming in macrophages that supports long-term suppression of inflammation
Source: PLoS Pathog. 2023 Dec 29;19(12):e1011886. doi: 10.1371/journal.ppat.1011886 (PMC10783791; doi:10.1371/journal.ppat.1011886)

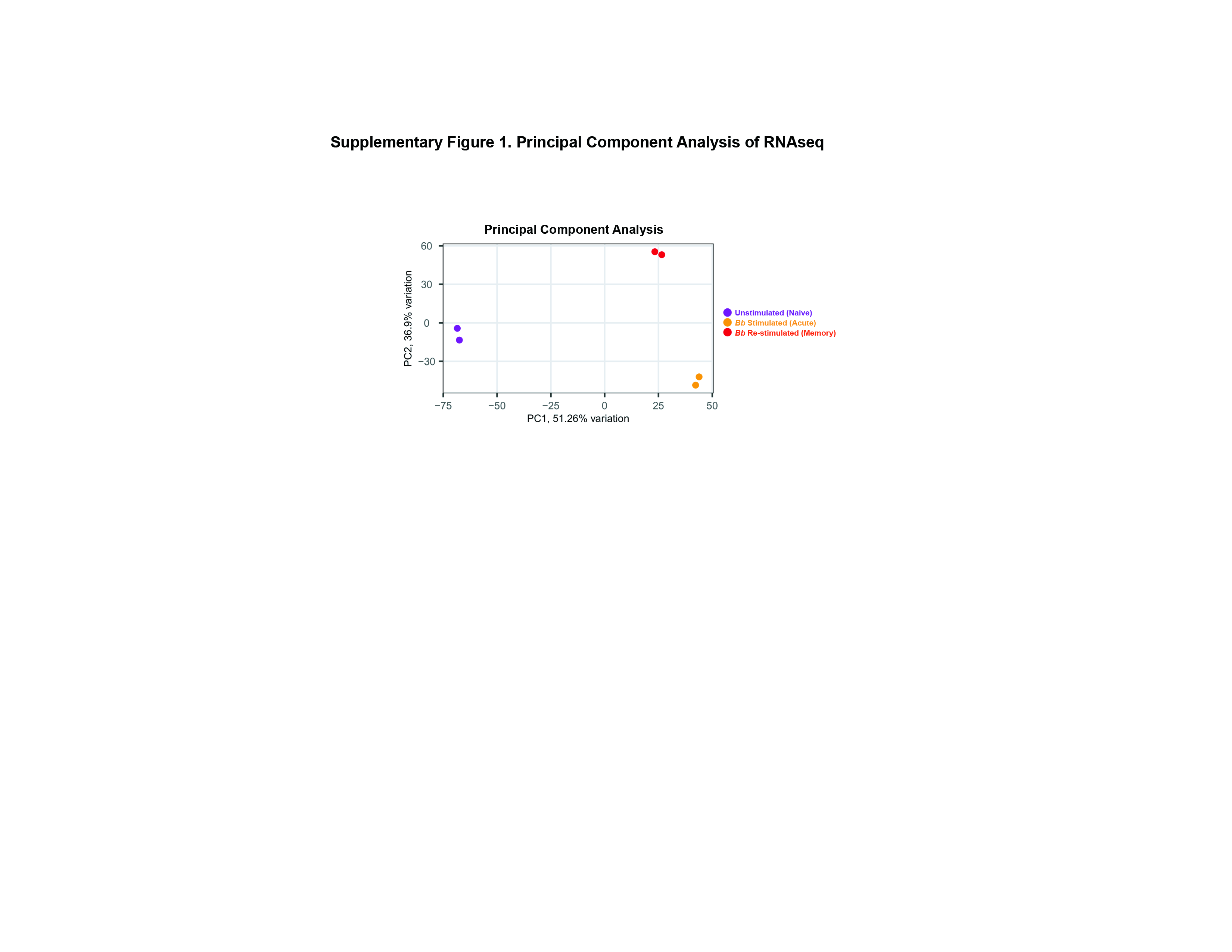

Supplement: S1 Fig — Principal component analysis of RNAseq data. Samples are colored by treatment of BMDMs: none (n = 2), stimulation (n = 2), re-stimulation (n = 2). (TIFF) [file ppat.1011886.s003.tiff]

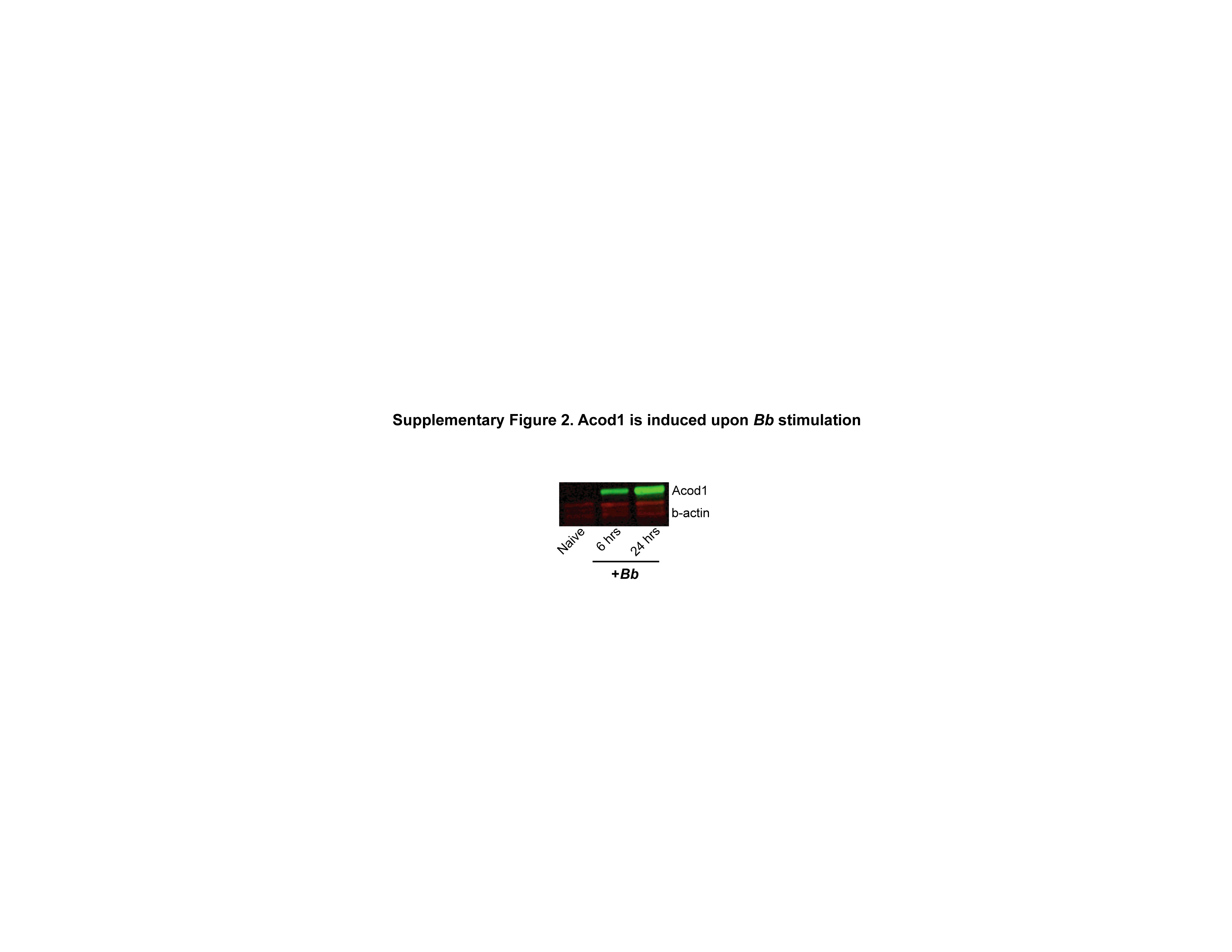

Supplement: S2 Fig — BMDMs were stimulated with Bb (MOI 10) for 0, 6 and 24 hours. Cells were collected and protein expression levels of Acod1 were assessed by immunoblot with β-actin as a loading control. (TIFF) [file ppat.1011886.s004.tiff]

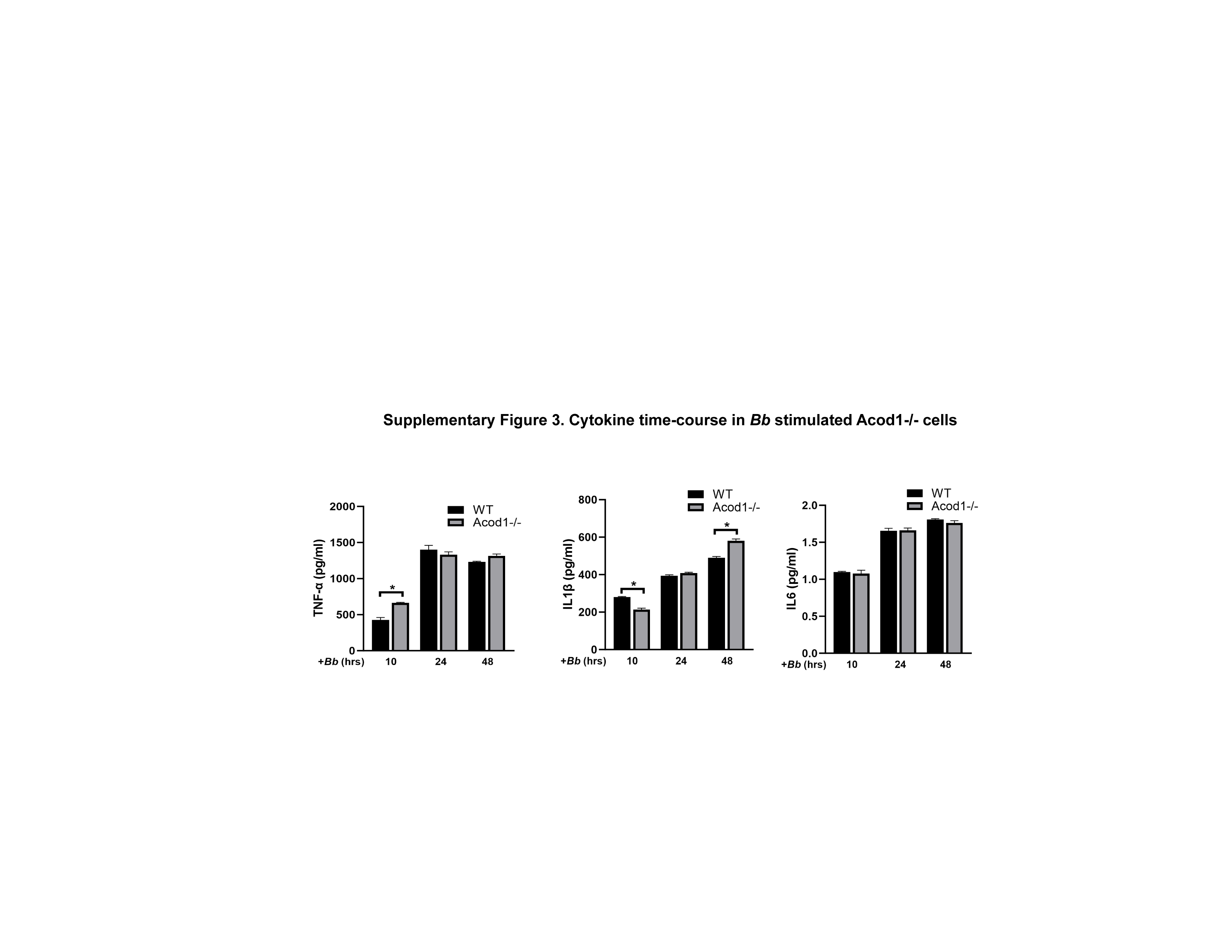

Supplement: S3 Fig — Bone marrow derived macrophage from NJ WT and Acod1 deficient mice were stimulated with Bb MOI10 for 10, 24 and 48 hours. Supernatants were collected at each time point and cytokines measured by ELISA. Graphed are two independent experiments and statistical significance assessed by unpaired T test, *p<0.05. (TIFF) [file ppat.1011886.s005.tiff]
